# Combination of Chromatographic Analysis and Chemometric Methods with Bioactivity Evaluation of the Antibacterial Properties of *Helichrysum italicum* Essential Oil

**DOI:** 10.3390/antibiotics13060499

**Published:** 2024-05-28

**Authors:** Tijana Zeremski, Olja Šovljanski, Vladimir Vukić, Biljana Lončar, Milica Rat, Nataša Perković Vukčević, Milica Aćimović, Lato Pezo

**Affiliations:** 1 Institute of Field and Vegetable Crops, 21000 Novi Sad, Serbia; tijana.zeremski@ifvcns.ns.ac.rs (T.Z.); acimovicbabicmilica@gmail.com (M.A.); 2 Faculty of Technology, University of Novi Sad, 21000 Novi Sad, Serbia; oljasovljanski@uns.ac.rs (O.Š.); vukicv@uns.ac.rs (V.V.); cbiljana@uns.ac.rs (B.L.); 3 Faculty of Sciences, University of Novi Sad, 21000 Novi Sad, Serbia; milica.rat@dbe.uns.ac.rs; 4 National Poison Control Centre, Military Medical Academy, 11000 Belgrade, Serbia; natavuk67@gmail.com; 5 Faculty of Medicine of the Military Medical Academy, University of Defense, 11042 Belgrade, Serbia; 6 Institute of General and Physical Chemistry, University of Belgrade, 11158 Belgrade, Serbia

**Keywords:** immortelle, *Helichrysum italicum*, neryl acetate, *γ*-curcumene, *ar*-curcumene, *α*-pinene, artificial neural network modeling

## Abstract

*Helichrysum italicum* (immortelle) essential oil is one of the most popular essential oils worldwide and it has many beneficial properties, including antimicrobial. However, in this plant, the chemical diversity of the essential oil is very pronounced. The aim of this work was to process the GC-MS results of four samples of *H. italicum* essential oil of Serbian origin by chemometric tools, and evaluate the antimicrobial activity in vitro and in silico. Overall, 47 compounds were identified, the most abundant were *γ*-curcumene, *α*-pinene, and *ar*-curcumene, followed by *α*-ylangene, neryl acetate, *trans*-caryophyllene, italicene, *α*-selinene, limonene, and italidiones. Although the four samples of *H. italicum* essential oil used in this study were obtained from different producers in Serbia, they belong to the type of essential oil rich in sesquiterpenes (*γ*-curcumene and *ar*-curcumene chemotype). In vitro antimicrobial potential showed that five were sensitive among ten strains of tested microorganisms: *Staphylococcus aureus*, *Listeria monocytogenes*, *Bacillus cereus*, *Saccharomyces cerevisiae,* and *Candida albicans*. Therefore, these microorganism models were used further for in silico molecular docking through the mechanism of ATP-ase inhibitory activity. Results showed that among all compounds from *H. italicum* essential oil, neryl acetate has the highest predicted binding energy. Artificial neural network modeling (ANN) showed that two major compounds *γ*-curcumene and *α*-pinene, as well as minor compounds such as *trans*-*β*-ocimene, terpinolene, terpinene-4-ol, isoitalicene, italicene, *cis*-*α*-bergamotene, *trans*-*α*-bergamotene, italidiones, *trans*-*β*-farnesene, *γ*-selinene, *β*-selinene, *α*-selinene, and guaiol are responsible for the antimicrobial activity of *H. italicum* essential oil. The results of this study indicate that *H. italicum* essential oil samples rich in *γ*-curcumene, *α*-pinene, and *ar*-curcumene cultivated in Serbia (Balkan) have antimicrobial potential both in vitro and in silico. In addition, according to ANN modeling, the proportion of neryl acetate and other compounds detected in these samples has the potential to exhibit antimicrobial activity.

## 1. Introduction

Essential oils, as a mixture of liposoluble volatile organic compounds, are characterized by a specific fragrance and numerous biological activities [1]. Interest in their use as natural sources of antioxidant and antimicrobial agents is constantly growing [2,3]. As alternative sources of antimicrobials, essential oils are used as preservations in food [4,5,6], cosmetics [7,8], and pharmaceutics [9,10]. Additionally, they are very important as flavoring agents [11]. Moreover, over the last two decades, essential oils have become popular in aromatherapy [12,13]. In agricultural practice, essential oils are used as biopesticides, for controlling plant pathogens and weeds [14,15], and in storages as fumigants and germination inhibitors suitable for organic production [16,17]. All these characteristics indicate that essential oils are versatile with a wide range of biological activity, and possess multipurpose applications.

*Helichrysum italicum* or immortelle essential oil is popular worldwide because of its many properties, such as antimicrobial, antioxidant, anti-inflammatory, anticarcinogenic, antidiabetic, and insecticidal [18,19]. Essential oil is obtained mainly by steam distillation of aboveground flowering parts of wild-growing or cultivated plants [20]. Considering the limited area in the natural distribution of *H. italicum* (Mediterranean and southeastern Europe), cultivation has been extended to continental Europe, mainly the Balkan Peninsula [21,22,23]. Due to the large number of subspecies and genotypes, together with the influence of environmental conditions, climate, and soil type, there is a significant variation in the essential oil quality [24,25,26]. Although *H. italicum* essential oil is highly valued, there is no official quality assessment standard. However, there are two main types of *H. italicum* essential oil on the market: Corsica (rich in oxygenated monoterpenes, especially neryl acetate) and Balkan (with dominant sesquiterpenes *γ*-curcumene and *ar*-curcumene and monoterpene *α*-pinene) [27]. The Corsica type of oil has been favored in the perfumery and cosmetic industry, while the Balkan type of quality has appeared during the last two decades with the expansion of *H. italicum* cultivation outside its native distribution range, on the continental territory of the Balkan Peninsula [28,29].

It is a well-known fact that the biological activity of this essential oil depends on its chemical composition [30,31,32]. As the polymorphism within the *H. italicum* species is very pronounced, and there is no official standard for quality assessment, it is essential to determine which components from the essential oil of *H. italicum* are responsible for its bioactivity. Previous research conducted with different commercial samples of *H. italicum* essential oil indicated that the antimicrobial and anti-inflammatory activities of the oil are linked with major and minor constituents, as well as their synergistic activity [33].

This study aimed to evaluate the chemical composition of four samples of *H. italicum* essential oil obtained from agricultural producers in Serbia, which grow this plant on a large scale and have their own distillation units. Additionally, to predict the efficiency of the essential oil compounds responsible for the antimicrobial activity, the following tasks were performed: assessment of the antimicrobial activity in vitro using the disk diffusion method and ten ATCC strains; in silico molecular modeling simulations using histidine kinase as antimicrobial target molecules; and modeling by applying an artificial neural network.

## 2. Results

### 2.1. Chemical Composition

Overall, 47 compounds were identified in *H. italicum* essential oil samples from Serbia, accounting for 93.04 and 97.13% of the whole essential oil composition (Table 1). The most abundant compounds were *γ*-curcumene (13.11–19.98%), *α*-pinene (9.75–14.45%), and *ar*-curcumene (5.8–14.0%), followed by *α*-ylangene (3.53–9.84%), neryl acetate (3.10–9.38%), *trans*-caryophyllene (3.59–6.00%), italicene (3.42–5.01%), α-selinene (2.61–5.72%), and limonene (1.96–5.29%).

**Figure 1:**
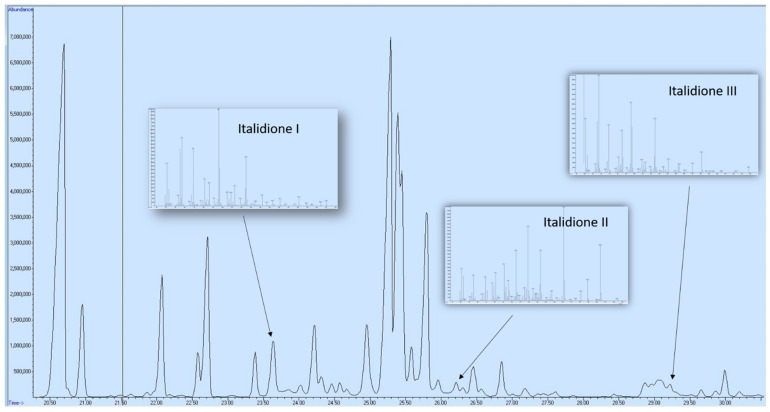
GS-MS chromatogram of *H. italicum* essential oil with respective italidiones MS spectra.

In all four samples of the essential oil of *H. italicum*, the dominance of sesquiterpenes is noticeable (55.14–66.68%). The second group in terms of representation was monoterpenes (16.82–26.00%), followed by esters (4.42–12.42) and ketones (5.05–7.50%). A class of compounds known as italidiones, *β*-diketones specific for *H. italicum*, was also present in the samples, and their mass spectra are shown in Figure 1.

**Figure 2:**
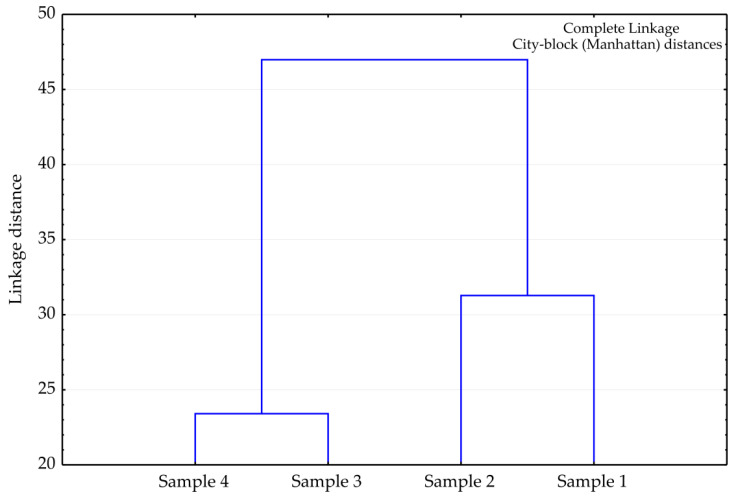
The cluster of the observed samples of *H. italicum* essential oil (according to Table 1).

The cluster analysis revealed two clusters: the first cluster includes samples 3 and 4, while the second encompasses samples 1 and 2 (Figure 2). The gained linkage distance between these clusters reached the value of almost 47. In summary, all four tested samples of *H. italicum* essential oils from Serbia belong to the typical Balkan type of oil, which is rich in γ-curcumene (between 13.11 and 19.8%). However, samples 1 and 2 are close to the Corsican type with slightly higher percentage of neryl acetate (7.42–9.38%), compared to its content in samples 3 and 4, (3.10–3.45%). Moreover, samples 3 and 4 contain significantly more *ar*-curcumene (11.74–14.40%) in comparison to samples 1 and 2 (5.91–9.08%).

### 2.2. Antimicrobial Potential of H. italicum Essential Oils

Table 2 provides insight into the antimicrobial potential of *H. italicum* essential oil against different microorganisms, measured by inhibition zones in millimeters. The data include bacterial, yeast, and fungal strains. In brief, *S. aureus* showed sensitivity to all four essential oil samples with varying inhibition zones. Sample 3 exhibited the highest activity (19.67 mm), followed by sample 1 (17.67 mm), sample 2 (12.00 mm), and sample 4 (10.00 mm). On the other hand, *B. cereus* responded to three *H. italicum* essential oil samples, but an antimicrobial effect was absent for sample 2. *L. monocytogenes* showed sensitivity to all samples, with sample 3 showing the highest activity (17.00 mm). *Escherichia coli*, *P. aeruginosa*, and *S.* Typhimurium showed no response to any of the samples.

As yeast representatives, *S. cerevisiae* and *C. albicans* showed zones of inhibition for all samples, with sample 2 displaying the highest activity against *S. cerevisiae* (18.67 mm), while sample 3 was the most effective against *C. albicans* (21.33 mm). This indicates that the samples have antifungal properties, with varying effectiveness. On the other hand, the oil samples showed exhibited no inhibition against *P. aurantiogriseum* or *A. brasiliensis*.

The antimicrobial potential of the samples varies significantly across different microbial strains. Sample 3 generally exhibited the highest antimicrobial activity among the samples tested, particularly against *S. aureus*, *B. cereus*, and *L. monocytogenes*, as well as *C. albicans* among the fungal strains. At the same time, the samples lack activity against certain strains (e.g., *E. coli*, *P. aeruginosa*, *S.* Typhimurium, *P. aurantiogriseum*, and *A. brasiliensis*).

### 2.3. In Silico Molecular Simulation Model

**Figure 3:**
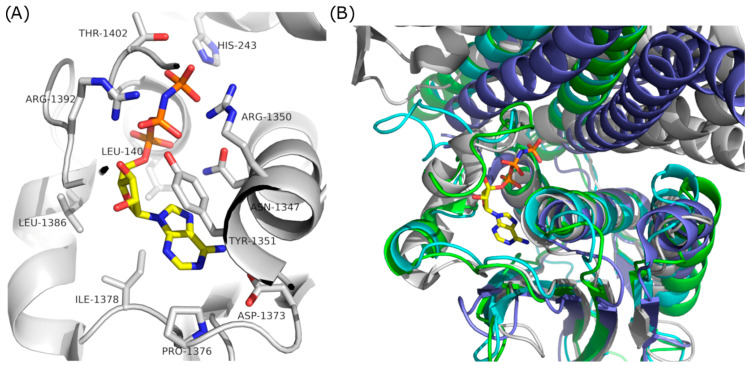
(**A**) Crystal structure of histidine kinase EnvZ from *E. coli* in complex with adenyl imidodiphosphate (PDBID: 4KP4); (**B**) aligned structures of catalytic histidine kinase domains of KdpD histidine kinase from *S. aureus* and *L. monocytogenes* with homologue histidine kinase EnvZ from *E. coli* (PDBID: 4KP4) and histidine kinase SrrB from *S. aureus* (PDBID: 6OAJ).

To investigate the potential mechanism of ATP-ase inhibitory activity and consequently the antimicrobial activity against *S. aureus* and L. monocytogenes, a structural evaluation was performed for the different samples of the studied essential oils from *H. italicum* to inhibit a well-known antimicrobial drug target KdpD histidine kinase. To verify the structures of KdpD histidine kinases from *S. aureus* and L. monocytogenes and to identify the binding site, their structures and sequences were compared with histidine kinase EnvZ from *E. coli* (PDBID: 4KP4) and histidine kinase SrrB from *S. aureus* (PDBID: 6PAJ), identified by a BLAST search for homologue sequences against the PDB database. Analysis of crystal structures of histidine kinase EnvZ from *E. coli* and histidine kinase SrrB from *S. aureus* identified active sites for ATP binding and catalysis (Figure 3A). The modeled structures of catalytic histidine kinase domains of KdpD histidine kinase from *S. aureus* and *L. monocytogenes* have high structural similarity with homologue histidine kinases from *E. coli*. In addition, regions in the downloaded Alpha Fold structures that are involved in ATP binding have very high model confidence (pLDDT > 70), which makes these structures appropriate for further analysis and molecular docking simulations (Figure 3B).

**Figure 4:**
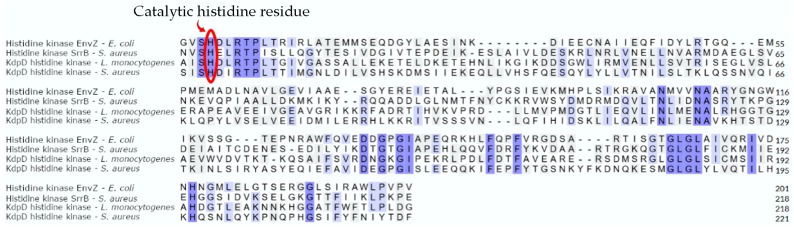
Sequence alignment of histidine kinase domains of KdpD histidine kinase from *S. aureus* and *L. monocytogenes* with homologue histidine kinase EnvZ from *E. coli* and histidine kinase SrrB from *S. aureus*.

In histidine kinase EnvZ from *E. coli*, His243 is a catalytic residue (Figure 3A). During the catalytic reaction, the active histidine residue assumes its position due to conformational changes induced by ATP binding. By sequence and structural alignment in catalytic histidine kinase domains of KdpD histidine kinase from *S. aureus* and *L. monocytogenes* with homologue histidine kinase EnvZ from *E. coli* and histidine kinase SrrB from *S. aureus*, His663 in *S. aureus* and His678 in *L. monocytogenes* were identified as catalytic residues (Figure 4).

**Figure 5:**
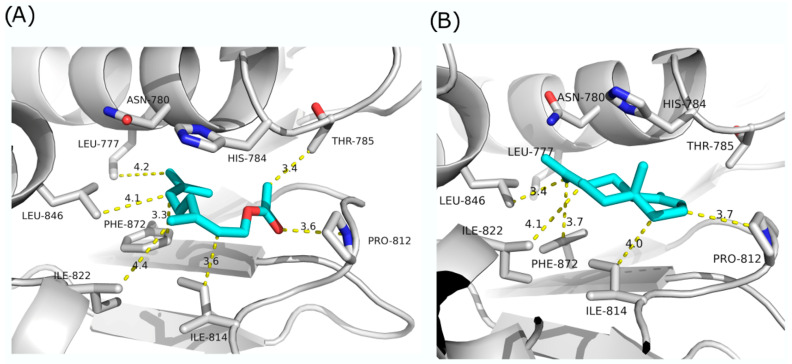
Molecular docking simulations of KdpD histidine kinase from *S. aureus* with (**A**) neryl acetate; (**B**) α-selinene.

Percentage of homology among the examined enzymes was in the range of 19.7 (between histidine kinase EnvZ—*E. coli* and KdpD histidine kinase—*S. aureus*) to 28.9 (between KdpD histidine kinase—*L. monocytogenes* and KdpD histidine kinase—*S. aureus*). Molecular docking simulation of KdpD histidine kinase from *S. aureus* with compounds identified in our essential oil samples revealed several potential candidates for binding and inhibition (Figure 5).

Among the compounds with high concentrations in samples, neryl acetate has the highest predicted binding energy (−46.03 kcal/mol) (Figure 5A). Its hydrophobic tail is buried among the hydrophobic residues Leu777, Leu846, Phe872, Ile822, and Ile814, while its polar head can make polar interactions with Pro812. In addition, the head is stabilized through hydrophobic interaction with Thr785. Another compound identified in our samples with favorable predicted binding energy is *α*-selinene (−37.69 kcal/mol) (Figure 5B). It does not have polar groups and makes hydrophobic interactions with Leu846, Phe872, Ile822, Ile814, and Pro812.

**Figure 6:**
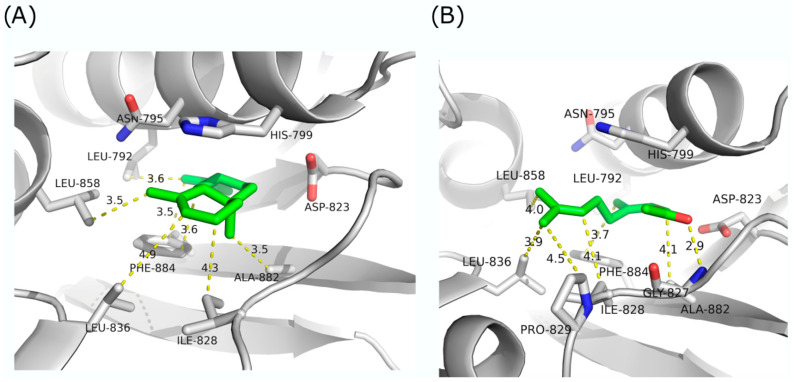
Molecular docking simulations of KdpD histidine kinase from *L. monocytogenes* with (**A**) α-selinene; (**B**) neryl acetate.

Analysis of KdpD histidine kinase from *L. monocytogenes* through molecular docking simulations identified the same candidates for binding and inhibition as in KdpD histidine kinase from *S. aureus* (Figure 6).

According to the presented simulation, α-selinene has the highest predicted binding energy against KdpD histidine kinase from *L. monocytogenes* (−37.89 kcal/mol) (Figure 6A). According to the results, hydrophobic interactions can occur with Leu792, Leu858, Leu836, Phe884, Ile828, and Ala 882. Similar to α-selinene, neryl acetate can bind to the hydrophobic pocket (−37.85 kcal/mol), making hydrophobic interactions with Leu858, Leu836, Phe884, Ile828, Ala 882, and Pro829 (Figure 6B). Furthermore, it can make polar interactions with Gly827. In contrast to α-selinene, neryl acetate is unable to bind deep into the pocket and interact with Leu792.

### 2.4. Artificial Neural Network Modeling

The optimized artificial neural network (ANN) model obtained demonstrated its capability to effectively predict the antimicrobial potential of *H. italicum* essential oil samples (expressed by *S. aureus*, *B. cereus*, *L. monocytogenes*, *S. cerevisiae*, and *C. albicans*), as shown in Table 1. The ideal configuration comprised a network architecture of 46 input, 24 hidden, and 5 output neurons in the multilayer perceptron (MLP), yielding the highest coefficient of determination (r^2^) values, reaching 1.000 during the training phase.

## 3. Discussion

In the Balkan type of oil, *H. italicum* essential oil is referred to as having *ar*- and *γ*-curcumene as its main compounds, which contribute to its special fragrance—strong, spicy, and herbal aroma with a honey-like nuance, often described as a curry-like odor [34,35,36]. On the other side, in the Corsica type of *H. italicum* essential oil, the most important compound is neryl acetate, which gives a specific sweet, floral fragrance, reminiscent of oranges and roses [36]. The presence of italidiones, β-diketones specific for the *H. italicum* essential oil, were characterized as early as 1967 [37,38], but there are still a limited number of papers that refer to their presence in the oil or their biological activity.

It is well known that *H. italicum* grows on alkaline, dry, sandy, and low-fertility soil. Samples 1 and 2 were obtained from producers located in Central and South Serbia. This is mountainous area, with a characteristic soil type and climate. Soils in Central Serbia are mostly classified as vertisols (according to World Reference Base for Soil Resources taxonomy) with a heavy texture and high clay content. In contrast, soil in South Serbia where H. italicum was sampled is classified as rendzina, which is typical in karst and mountain landscapes, where carbonate-rich material occurs on slopes. Soils in North Serbia mostly belong to chernozem type, a very fertile soil that can produce high agricultural yields with its high moisture-storage capacity. From the other side, samples 3 and 4 were obtained from producers located in North Serbia. This is a lowland region with a fertile chernozem soil type. In addition to the genotype, the soil and microclimate certainly have a significant influence on the stability in the quality of the essential oil [39,40].

Experimental results provided in Table 2 indicate that samples 1 through 4 had varying levels of antimicrobial activity against certain bacterial strains (e.g., *S. aureus*, *B. cereus*, *L. monocytogenes*). However, they showed no activity against *E. coli*, *P. aeruginosa*, or *S.* Typhimurium. The variation in antimicrobial activity among the examined samples and the previously reported studies can be due to several factors, including the specific strains tested (clinical isolates or standard reference microorganisms), the concentration of essential oil or its components, the methodologies used, and the experimental conditions. The primary components (neryl acetate, *α*-pinene, *γ*-curcumene) are known for their antimicrobial properties, suggesting that the presence and concentration of these compounds significantly influence the overall antimicrobial efficacy of *H. italicum* essential oil. Different methodologies (e.g., agar well diffusion, broth microdilution, microdilution assay) can yield varying results due to differences in sensitivity, concentration, and interaction dynamics between the essential oil and the microbial cells. Given correlations between samples and their antimicrobial potential, this quantitative analysis of the biocide effect revealed variations in the concentrations of major compounds such as *γ*-curcumene, *α*-pinene, and neryl acetate across the samples. For instance, sample 1 demonstrated a higher content of α-pinene (14.45%) than sample 4 (11.21%), which corresponded with its enhanced antimicrobial efficacy against *S. aureus*. The detailed chemical compositions are presented in Table 2, highlighting these differences. Furthermore, antimicrobial testing showed that sample 3 exhibited the strongest activity against *L. monocytogenes*, which could be correlated with its elevated levels of neryl acetate (9.38%). The inhibition zone measurements for each sample against various microorganisms provide a clear comparison of their antimicrobial potentials.

Analyzing all available results for the antimicrobial activity of *H. italicum* essential oil offers an understanding of microbial responses and suggests avenues for future research. The observed patterns in microbial behavior during contact with the essential oil and potential directions for antimicrobial investigation emerge when considering the variability in antimicrobial efficacy observed across different microbial strains and the chemical compositions of the tested samples. For example, a comparison of the results from Table 2 defines strong strain-specific connections, indicating that microbial sensitivity to *H. italicum* essential oil and its components varies significantly among different strains. For instance, *S. aureus* frequently shows sensitivity to *H. italicum* essential oil, suggesting a particular vulnerability of Gram-positive bacteria to the essential oil’s components [41]. Conversely, *E. coli* and *P. aeruginosa* often exhibit resistance, highlighting a potential pattern where certain Gram-negative bacteria may have inherent or developed resistance mechanisms against the components found in *H. italicum* essential oil [42]. Another observation can be directed to component-specific activity where antimicrobial potential appears to be closely tied to its chemical composition, particularly the concentrations of neryl acetate, *α*-pinene, and *γ*-curcumene. These compounds have been repeatedly associated with antimicrobial efficacy, suggesting their relative abundance may directly influence the essential oil’s overall antimicrobial potential [33,43].

Further research should aim to elucidate the specific mechanisms through which *H. italicum* essential oil and its primary components exert antimicrobial effects. Understanding these mechanisms at the molecular level can help in modifying essential oil formulations for targeted antimicrobial applications. Exploring the synergistic effects of *H. italicum* essential oil components with conventional antibiotics or other antimicrobial oils could open new directions for combination therapies that enhance antimicrobial efficacy.

In summary, identifying a range of compounds commonly associated with antimicrobial properties in the tested samples, including *γ*-curcumene, *α*-pinene, and neryl acetate, showed that the relative concentrations of these compounds varied notably among the samples. For example, sample 1 was characterized by a higher concentration of *α*-pinene, which correlated with its stronger antimicrobial activity against *S. aureus* (Table 2). This observation aligns with the literature suggesting that *α*-pinene has significant antibacterial properties [44]. In contrast, sample 3, which showed the most substantial activity against *L. monocytogenes*, had elevated levels of neryl acetate, a compound known for its efficacy against Gram-positive bacteria [45]. The observed variability in chemical profiles among the oil samples can be attributed to several environmental and procedural factors. Differences in soil composition, climate conditions, plant phenotype, and extraction methods are known to affect the secondary metabolite profiles in plants [46]. For instance, the variation in *γ*-curcumene levels between samples 2 and 4 may be due to differences in the distillation processes or harvest times, which warrants further investigation.

Additionally, some specifications for the sensitivity of tested microorganisms can be defined. Namely, antimicrobial testing revealed significant variability in the effectiveness of the essential oils against different microbial targets. While the essential oils demonstrated potent activity against Gram-positive bacteria, they exhibited reduced efficacy against Gram-negative bacteria. This pattern was consistent across all tested oil samples. The enhanced susceptibility of Gram-positive bacteria to essential oils can be attributed to their thicker peptidoglycan layer, which lacks an additional outer membrane barrier. This structural difference makes them more sensitive to the hydrophobic compounds found in essential oils that can disrupt lipid bilayers [47]. In contrast, the resistance of Gram-negative bacteria is likely due to their outer membrane, which is rich in lipopolysaccharides. This membrane acts as an effective barrier against many hydrophobic substances, limiting the penetration of antimicrobial agents. The distinct cell wall composition of fungi and yeasts, primarily composed of chitin and glucans, also provides different challenges and interactions with the bioactive compounds in essential oils [46].

The next step in this study involves the prediction of antimicrobial activity mechanism through the in silico molecular simulation models by investigating the ATP-ase inhibitory activity of *H. italicum* essential oils. Namely, the antimicrobial activity of essential oils through ATP-ase inhibitory activity has been described in numerous research reports [48,49,50]. Also, literature data shows decreased ATP-ase activity inside *S. aureus* when treated with some of the essential oil components (thymol and carvacrol) [51]. Thymol and carvacrol can affect cell membrane permeability and the ATP-ase activity of *S. aureus*, thus inhibiting its metabolic activity and growth. Similar to this is the case of *L. monocytogenes* [52]. Inspired by these facts, this study investigated the connection between the chemical content of *H. italicum* essential oils, their antimicrobial activity against selected microbial strains, and their possibilities to inhibit KdpD histidine kinase from *S. aureus* and *L. monocytogenes*, which regulates potassium homeostasis. ATP-ase, specifically KdpD histidine kinase, is a well-known antimicrobial target that regulates potassium homeostasis in bacteria such as *S. aureus* and *L. monocytogenes*, which have shown certain sensitivity in this study. The choice of ATP-ase inhibitory activity is inspired by reports in the literature showing that certain essential oil components, like thymol and carvacrol, can disrupt bacterial cell membrane permeability and ATP-ase activity, inhibiting metabolic activity and growth [51,52]. Furthermore, analysis of histidine kinase binding sites revealed hydrophobic residues in all analyzed proteins. A large hydrophobic pocket in the active site is perfectly shaped for essential oils that are mostly hydrophobic. In addition, some of the analyzed enzymes have polar groups that can bind polar residues, such as histidine and asparagine, or main chain polar groups. Sequence alignment of the analyzed sequences revealed that higher homology is observed in the regions involved in substrate binding as well as in the regions involved in the conformational change that occurs prior to the catalytic reaction. Most of the differences are reflected in the replacement of residues with similar characteristics (charged with charged, hydrophobic with hydrophobic, etc.). Structural analysis of the examined domains shows that they share a highly conserved helix-turn-helix fold. Similar results of sequence and structural alignments among KdpD histidine kinases from other microorganisms were obtained in previous research [53,54]. This data analysis demonstrates molecular docking simulations that compounds found in *H. italicum* essential oils have the potential to bind and inhibit KdpD histidine kinase, suggesting a mechanism through which tested oils exert their antimicrobial effects. Furthermore, the molecular docking simulations described in the research provide a detailed mechanism connecting the antimicrobial activity of *H. italicum* essential oils to their inhibitory action on ATP-ase, specifically the KdpD histidine kinase in *S. aureus* and *L. monocytogenes*.

The essential oils contain compounds identified through molecular docking simulations to bind with significant affinity to the KdpD histidine kinase, indicating their potential as effective inhibitors of this enzyme [55]. These compounds interact with the enzyme in a manner that highlights their hydrophobic and, in some cases, polar interactions with specific amino acid residues within the enzyme’s active site. In this study, neryl acetate and α-selinene demonstrated substantial predicted binding energies and interactions that suggest a strong inhibitory potential on the enzyme’s activity. Further experimental research with pure compounds would be beneficial to confirm these results. This inhibitory action is significant because the KdpD histidine kinase plays a crucial role in bacterial homeostasis and survival, particularly in the regulation of potassium homeostasis. By inhibiting this enzyme, the essential oils disrupt the bacterial cell’s regulatory mechanisms, leading to its inability to maintain essential cellular functions, which could ultimately result in bacterial death or significant inhibition of growth. The antimicrobial testing results presented in Table 2 provide empirical evidence supporting the theoretical predictions based on the molecular docking simulations. The essential oils exhibited varying degrees of antimicrobial activity against different bacterial and fungal strains, with some samples showing significant activity against *S. aureus*, *B. cereus*, and *L. monocytogenes*, and yeast strains such as *S. cerevisiae* and *C. albicans*. These results are particularly notable against the strains of *S. aureus* and *L. monocytogenes*, aligning with the in silico predictions of the essential oils’ inhibitory effects on the KdpD histidine kinase. This connection between the molecular docking simulations and antimicrobial testing results emphasizes the potential of *H. italicum* essential oils as a source for natural antimicrobial agents.

The complexity of the ANN model for predicting output variables was notable, featuring 1253 weight-bias coefficients, reflecting the significant nonlinearity of the observed system. A comparison between experimental and ANN-predicted values showed strong agreement, with the ANN values closely aligned with the measured values, particularly evident in the high r^2^ values. The sum of squared errors (SSE) obtained from the ANN model was comparable to experimental errors for output variables. Moreover, the lack-of-fit tests showed insignificance, affirming the model’s effectiveness in predicting output variables.

These results indicate that the high antibacterial activity in our samples against *S. aureus* and *L. monocytogenes* could have been induced by high concentrations of neryl acetate and α-selinene. Although both compounds perfectly fit the active site, only neryl acetate has the proposed characteristics for a good inhibitor; it has a polar part that provides specificity and a hydrophobic part that makes strong hydrophobic interactions.

### Global Sensitivity Analysis

**Figure 7:**
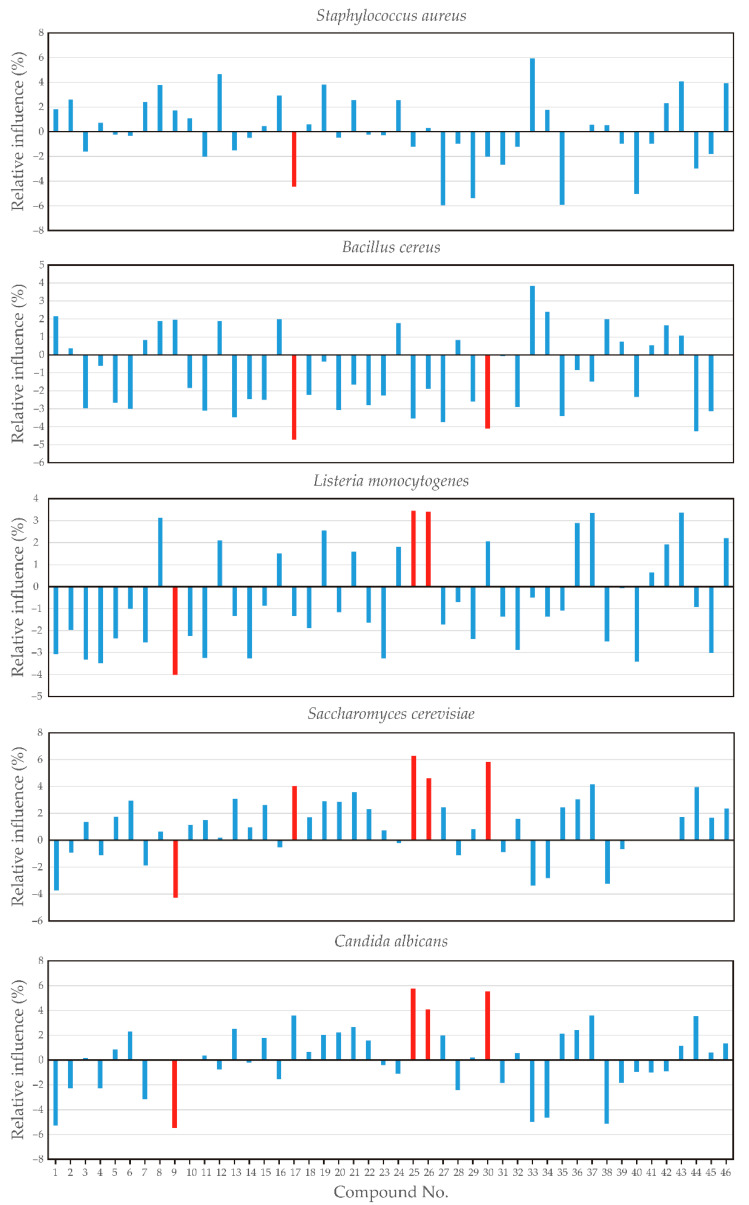
The relative importance of the antimicrobial potential of *H. italicum* essential oil samples (expressed by *S. aureus*, *B. cereus*, *L. monocytogenes*, *S. cerevisiae*, and *C. albicans*), according to the chemical composition of different samples (compound codes are presented in Table 1).

A global sensitivity analysis using Yoon’s interpretation method revealed the key influential parameters for predicting the antimicrobial potential of *H. italicum* essential oil samples, according to the chemical composition of these samples. According to global sensitivity analysis, the strongest influence on the antimicrobial potential of *H. italicum* on *S. aureus*, *B. cereus*, *L. monocytogenes*, *S. cerevisiae*, and *C. albicans* was obtained by *α*-pinene, *trans*-*β*-ocimene, terpinolene, terpinene-4-ol, isoitalicene, italicene, *cis*-*α*-bergamotene, *trans*-*α*-bergamotene, italidione I, *trans*-*β*-farnesene, *γ*-selinene, *γ*-curcumene, *β*-selinene, *α*-selinene, italdione II, italdione III, and guaiol (Figure 7). The influence of the most significant compounds such as *trans*-β-ocimene, terpinene-4-ol, isoitalicene, italicene, and italidione (Mw 210) on the relative importance of the antimicrobial potential of *H. italicum* is highlighted by red bars shown in Figure 7.

## 4. Materials and Methods

### 4.1. Essential Oil Samples

The essential oil used in this study was obtained directly from agricultural producers, who market the oils to trading companies. Samples 1 and 2 were obtained from producers located in Central and South Serbia. Samples 3 and 4 were obtained from producers located in North Serbia (Vojvodina Province).

### 4.2. Chromatographic Analysis of Essential Oil Samples

An Agilent 7890A chromatograph (Santa Clara, CA, USA) equipped with a flame ionization detector (FID) and HP-5 capillary column (30 m × 0.25 mm, film thickness 0.25 μm) was used for quantitative analysis of the essential oil. The oven temperature was set as follows: 60 °C, ramp rate of 3 °C/min, and final temperature of 240 °C held for 2 min. The injector and detector temperatures were 230 and 250 °C, respectively. The carrier gas was He with a flow rate of 1 mL/min.

Gas chromatography–mass spectrometry profiling of *H. italicum* essential oils was conducted using an Agilent 6890N GC coupled with a CTC Analytics CombiPal autosampler (Santa Clara, CA, USA), a nonpolar HP5-MS column, and an Agilent 5975B MS (Santa Clara, CA, USA) (details are given by Aćimović et al. [56]).

Quantitative results were obtained from GC-MS analyses. The individual peaks were identified by a computer matching of mass spectra with the ADAMS and NIST mass spectral database and by comparison of their linear retention indices (LRI) relative to a series of n-hydrocarbons (C9–C40). The percentage composition of compounds (relative quantity) in the essential oil and fraction of acids were computed from the GC-FID peak areas using the normalization method, without correction factors.

### 4.3. Antimicrobial Potential

To investigate the antimicrobial potential, various microorganisms were employed: *Escherichia coli*, *Pseudomonas aeruginosa*, and *Salmonella enterica* serovar Typhimurium as Gram-negative strains; *Staphylococcus aureus, Bacillus cereus*, and *Listeria monocytogenes* as Gram-positive strains; *Saccharomyces cerevisiae* and *Candida albicans* as yeasts; and fungal representatives *Aspergillus brasiliensis* and *Penicillium aurantiogriseum*. All tested strains are from the American Type Culture Collection (ATCC numbers are given in Table 2). The overnight cultures were prepared as follows:

1. The prokaryotes were streaked from −80 °C glycerol stock onto Müller–Hinton Agar (HiMedia, Mumbai, India) and incubated at 37 °C for 24 h.
2. The eukaryotes were streaked from −80 °C glycerol stock onto Sabouraud Maltose Agar (HiMedia, Mumbai, India) and incubated at 25 °C for 5 days (*A. brasiliensis and P. aurantiogriseum*), 30 °C for 48 h (*S. cerevisiae*), and 37 °C for 48 h (*C. albicans*).

The antimicrobial potential was examined by using the disk diffusion method, which was earlier described in detail by Aćimović et al. [57]. After incubation, all sensitive microorganisms showed a lack of growth (no appearance of micro- or macrocolonies) around a disk where an oil aliquot of 15 µL was applied. The inhibition zones obtained by this method were evaluated as the diameter of the illuminated zone, expressed in millimeters. All experimental measurements were performed in triplicate.

### 4.4. In Silico Molecular Modeling Simulations

Three-dimensional structures of KdpD histidine kinase from *S. aureus* and *L. monocytogenes* were retrieved from the AlphaFold database (AF-Q2FWH7 and AF- A0A0E1R9J8, respectively) [58]. The catalytic histidine kinase domains were used from *S. aureus* and *L. monocytogenes* (positions 660–880 and 675–892, respectively) for further analysis. The sequences and structures were compared and aligned with the histidine kinase domain of the sensor histidine kinase EnvZ from *E. coli* (UniProt: P0AEJ4) (PDBID: 4KP4) [59], (with a sequence identity of 23.98% with *S. aureus* and 28.93% with *L. monocytogenes*), as well as the sensor protein SrrB from *S. aureus* (UniProt: Q9L523) (PDBID: 6PAJ) [59], (with a sequence identity of 24.76% with *S. aureus* and 26.76% with *L. monocytogenes)*, which are homologous to the KdpD histidine kinase from *S. aureus* and *L. monocytogenes*. The structure of histidine kinase EnvZ from *E. coli* (PDBID: 4KP4) was used as it has ligand bonds in the active site, while the structure of sensor protein SrrB from *S. aureus* (PDBID: 6PAJ) was used as it is in the open position, which is suitable for initial ligand binding. The modeled structures of catalytic histidine kinase domains from *S. aureus* and *L. monocytogenes* were further minimized using the OPLS force field and the Maestro protein preparation workflow [60]. The protein structures were prepared for molecular docking simulations using the following parameters: polar hydrogen atoms were added, protonation types and termini treatment were enabled. The 3D structures of the examined volatile compounds from essential oil were built using the Maestro program in the Schrödinger package (Release 2024-2). The geometry optimizations of compounds used for molecular docking simulation and KdpD histidine kinases were performed using the OPLS4 force field and the Powell conjugated gradient algorithm method. The convergence criterion was set at 0.01 kcal/(mol Å) and a maximum iteration was set at 1000.

The molecular docking simulations were performed using the Glide 4.0 XP program [61], with extra precision mode and flexible ligand. Epik state penalties were used in calculating the docking score. Ligand binding affinities were calculated by the MM-GBSA method using the VSGB 2.0 solvation model [62]. The residues within a 4.0 Å distance from the ligand were assigned as flexible. The results obtained were visualized using the PyMol 3.0 program.

Previously conducted studies provided detailed insight into the activities of histidine kinase, and we used established principles for visualization and analysis [63,64].

### 4.5. Statistical Analysis

A three-layered multilayer perceptron (MLP) model was utilized for artificial neural network (ANN) analysis to assess the antimicrobial potential of *H. italicum* essential oil samples given in Table 2 (expressed by *S. aureus*, *B. cereus*, *L. monocytogenes*, *S. cerevisiae*, and *C. albicans*). Normalization of the experimental dataset was carried out to enhance the ANN performance. The Broyden–Fletcher–Goldfarb–Shanno (BFGS) algorithm was employed to solve nonlinear problems encountered during network modeling. Various network topologies, exceeding 100,000 in number, were explored by adjusting the number of neurons in the hidden layer (ranging from 5 to 20) and initializing weights and biases randomly [65].

A global sensitivity analysis was performed using Yoon’s interpretation method to assess the relative impact of bioactive compound content on antimicrobial activity, employing the weight coefficients derived from the ANN model [66].

Statistical analysis was conducted using StatSoft Statistica 10.0^®^ software.

## 5. Conclusions

This study elucidates the multifaceted properties and therapeutic potential of *Helichrysum italicum* essential oil. The findings highlight the importance in understanding the variability in essential oil composition, particularly in samples from different geographical regions like Serbia, and its impact on antimicrobial activity. The antimicrobial assays reveal varying degrees of activity against bacterial and fungal strains. Moreover, molecular docking simulations identified neryl acetate and α-selinene as promising inhibitors of KdpD histidine kinase, indicating potential mechanisms of action against pathogenic bacteria like *S. aureus* and *L. monocytogenes*. 

Overall, this study contributes to a deeper understanding of the biological activities and industrial potential of *H. italicum* essential oil, facilitating further research and application development in the field of natural products and alternative medicine.

## Figures and Tables

**Table 1:** Chemical composition (%) of four different samples of *H. italicum* essential oils from Serbia.

	Compound	Class	RT (min)	Sample 1	Sample 2	Sample 3	Sample 4
1	*α*-pinene	Monoterpene	4.935	14.45	9.75	11.65	11.21
2,3	*α*-fenchene + camphene	Monoterpene	5.191	0.53	0.38	0.33	0.18
4	*β*-pinene	Monoterpene	5.932	0.54	0.57	0.19	0.30
5	myrcene	Monoterpene	6.281	0.20	0.12	nd	nd
6	*α*-terpinene	Monoterpene	7.043	0.20	0.21	0.16	0.16
7	*p*-cymene	Monoterpene	7.285	0.27	0.32	0.22	0.21
8	limonene	Monoterpene	7.427	5.29	3.30	2.98	1.96
9	1,8-cineole	Monoterpene	7.484	0.31	0.29	0.56	0.32
10	*trans*-*β*-ocimene	Monoterpene	8.032	0.16	nd	nd	nd
11	isobutyl angelate	Ester	8.132	0.45	0.45	0.29	0.24
12	*γ*-terpinene	Monoterpene	8.388	0.48	0.50	0.33	0.39
13	terpinolene	Monoterpene	9.412	0.21	0.18	0.29	0.15
14	linalool	Monoterpene	9.839	1.35	2.24	0.47	0.70
15	2-methyl butyl-2-methyl butyrate	Ester	9.979	0.23	0.23	0.13	0.14
16	2,4, dimethyloctan, 3-5dion (Mw 170)	Ketone	11.878	1.32	1.53	1.07	0.92
17	borneol	Monoterpene	12.312	0.10	nd	0.27	0.10
18	terpinene-4-ol	Monoterpene	12.790	0.30	0.39	0.26	0.34
19	3,4 octan dione	Ketone	13.196	0.73	0.79	0.43	0.35
20	*α*-terpineole	Monoterpene	13.339	0.39	0.44	0.48	0.32
21	nerol	Monoterpene	14.877	1.22	1.82	0.54	0.48
22	3-Butyl-6-methylpiperazine-2,5-dione Mw 184, dione	Ketone	17.291	0.30	0.55	0.43	nd
23	neryl acetate	Ester	20.318	7.42	9.38	3.45	3.10
24	*α*-ylangene	Sesquterpene	20.618	9.84	9.61	3.53	4.00
25	*α*-copaene	Sesquterpene	20.960	2.64	1.67	4.94	3.04
26	isoitalicene	Sesquterpene	21.864	0.11	0.18	0.18	0.16
27	italicene	Sesquterpene	22.085	3.42	4.50	5.01	4.26
28	*cis*-*α*-bergamotene	Sesquterpene	22.605	1.11	1.39	0.99	1.72
29	*trans*-caryophyllene	Sesquterpene	22.748	4.82	3.59	5.55	6.00
30	*trans*-*α*-bergamotene	Sesquterpene	23.396	1.17	1.19	1.03	1.52
31	italidione I (Mw 210)	Ketone	23.681	1.90	3.84	2.96	2.89
32	*α*-humulene	Sesquterpene	24.037	0.24	0.17	0.26	0.36
33	neryl propanoate	Ester	24.215	2.12	2.36	0.60	0.94
34	*trans*-*β*-farnesene	Sesquterpene	24.314	0.47	nd	0.46	nd
35	*γ*-selinene	Sesquterpene	24.963	2.46	1.05	2.56	2.22
36	*γ*-curcumene	Sesquterpene	25.362	13.11	15.81	13.34	19.98
37	*ar*-curcumene	Sesquterpene	25.447	5.91	9.08	14.40	11.74
38	*β*-selinene	Sesquterpene	25.646	0.68	2.10	3.07	1.90
39	*α*-selinene	Sesquterpene	25.853	5.72	2.61	5.17	5.38
40	*α*-muurolene	Sesquterpene	25.995	0.35	nd	0.74	0.86
41	italdione II (Mw 224)	Ketone	26.238	0.26	0.18	nd	0.49
42	*γ*-cadinene	Sesquterpene	26.466	0.95	0.78	1.36	1.42
43	*δ*-cadinene	Sesquterpene	26.879	0.97	0.51	2.33	1.34
44	italdione III (Mw 238)	Ketone	28.880	0.54	0.61	0.98	0.47
45	guaiol	Sesquterpene	29.671	0.19	0.36	0.11	0.20
46	rosifoliol	Sesquterpene	30.005	0.73	0.80	0.33	0.47
47	*α*-eudesmol	Sesquterpene	31.544	0.25	0.30	0.38	0.11
	Total monoterpenes			26.00	20.51	18.73	16.82
	Total esters			10.22	12.42	4.47	4.42
	Total ketones			5.05	7.50	5.87	5.12
	Total sesquterpenes			55.14	55.70	65.74	66.68
	TOTAL IDENTIFIED			96.41	97.13	94.81	93.04

RT—retention time; nd—not detected.

**Table 2:** Assessment of the antimicrobial potential of *H. italicum* essential oil samples from Serbia measured as inhibition zones (mm).

		Samples				Controls		
						Positive Control for Bacteria	Positive Control for Yeast and Fungi	Negative Control
ATCC	Microbial Strain	1	2	3	4	Cefotaxime + Clavulanic Acid	Actidione	Water
25922	*Escherichia coli*	nd	nd	nd	nd	27.00	/	nd
27853	*Pseudomonas aeruginosa*	nd	nd	nd	nd	21.00	/	nd
13311	*Salmonella* Typhimurium	nd	nd	nd	nd	29.00	/	nd
25923	*Staphylococcus aureus*	17.67	12.00	19.67	10.00	28.33	/	nd
11778	*Bacillus cereus*	11.00	nd	12.67	7.33	27.00	/	nd
19115	*Listeria monocytogenes*	7.33	13.00	17.00	13.00	29.00	/	nd
9763	*Saccharomyces cerevisiae*	13.67	18.67	15.33	14.67	/	34.00	nd
10231	*Candida albicans*	7.67	13.67	11.33	10.33	/	37.00	nd
16025	*Penicillium aurantiogriseum*	nd	nd	nd	nd	/	26.33	nd
16404	*Aspergillus brasiliensis*	nd	nd	nd	nd	/	27.00	nd

nd—not detected.

## Data Availability

The original contributions presented in the study are included in the article; further inquiries can be directed to the corresponding author.
